# Supplementary figures and images for: The effectiveness of Evodia rutaecarpa hot compress on the recovery of gastrointestinal function after laparoscopic surgery for colorectal cancer: A propensity score-matched retrospective cohort study
Source: PLoS One. 2025 Feb 20;20(2):e0303951. doi: 10.1371/journal.pone.0303951 (PMC11841865; doi:10.1371/journal.pone.0303951)

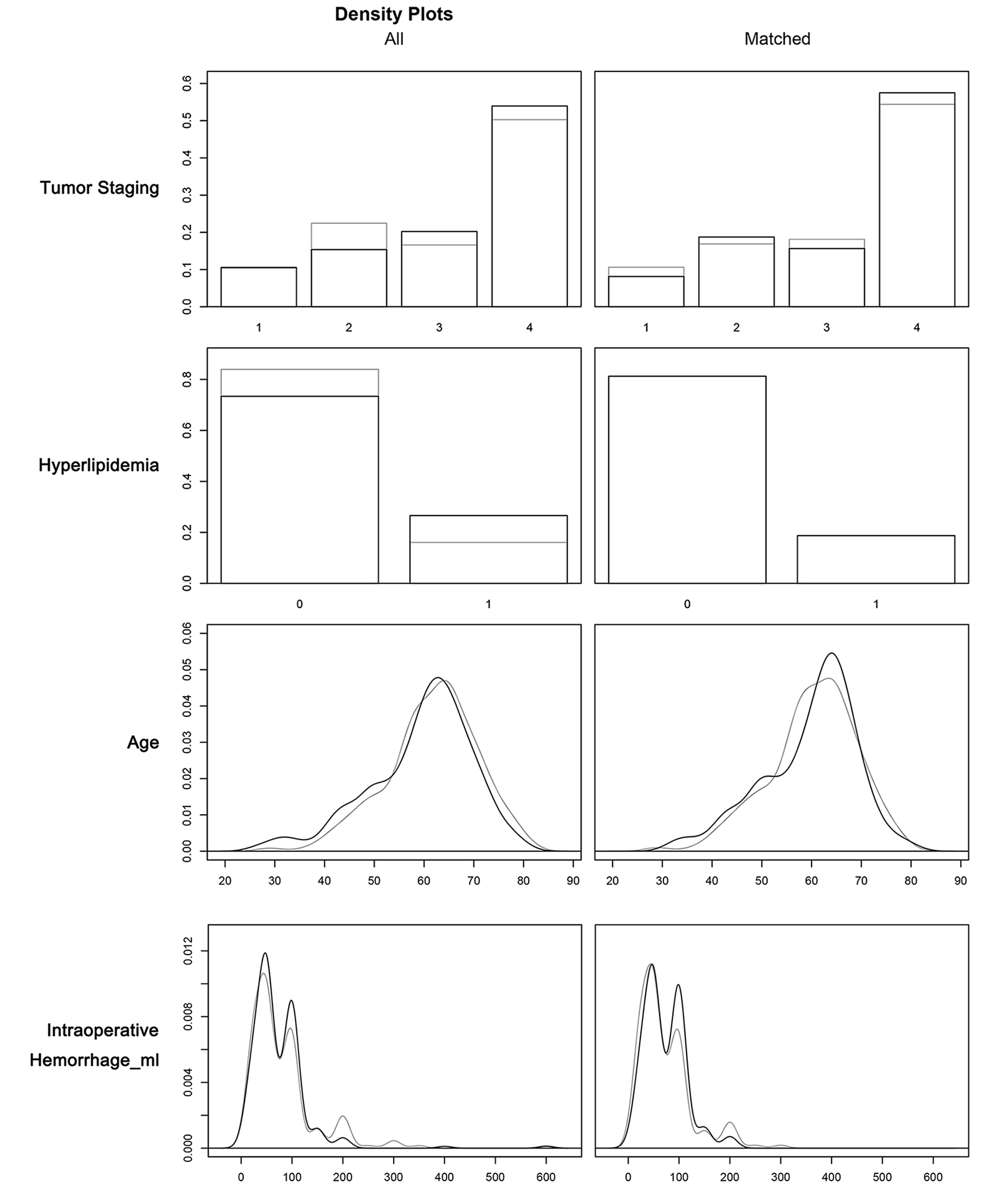

Supplement: S1 Fig — (TIF) [file pone.0303951.s001.tif]

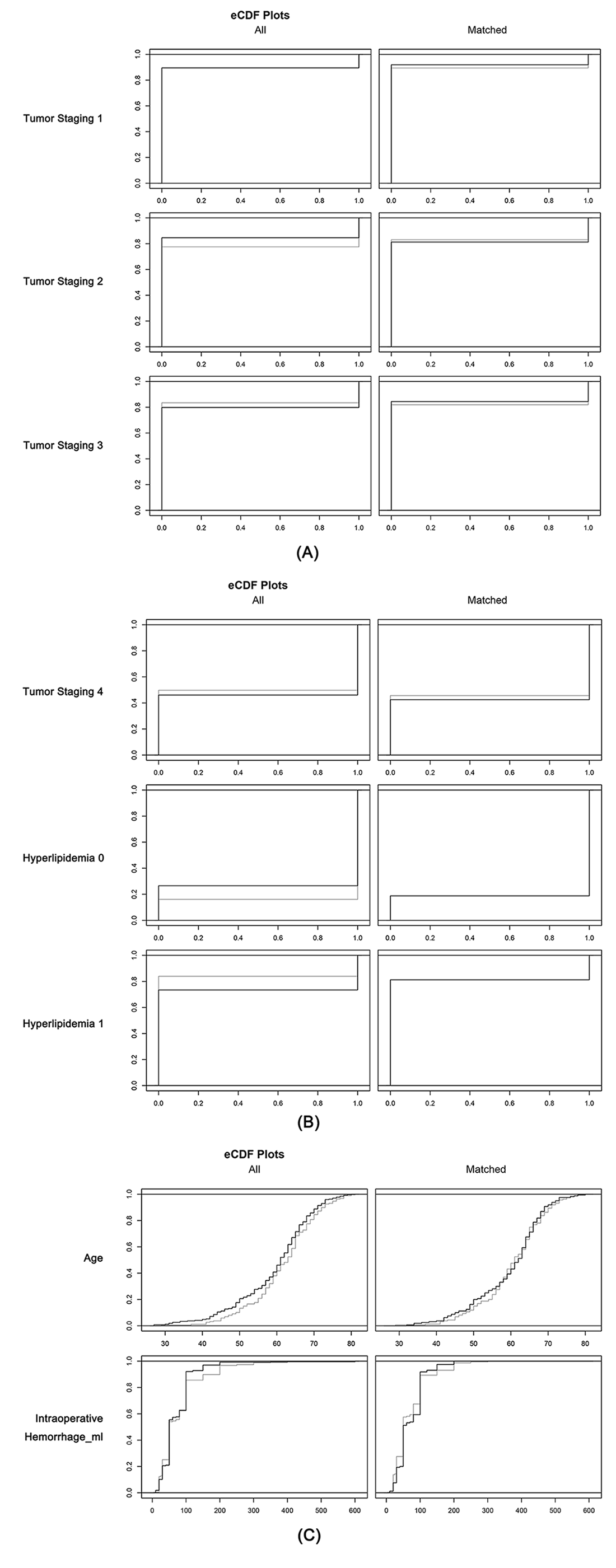

Supplement: S2 Fig — (TIF) [file pone.0303951.s002.tif]

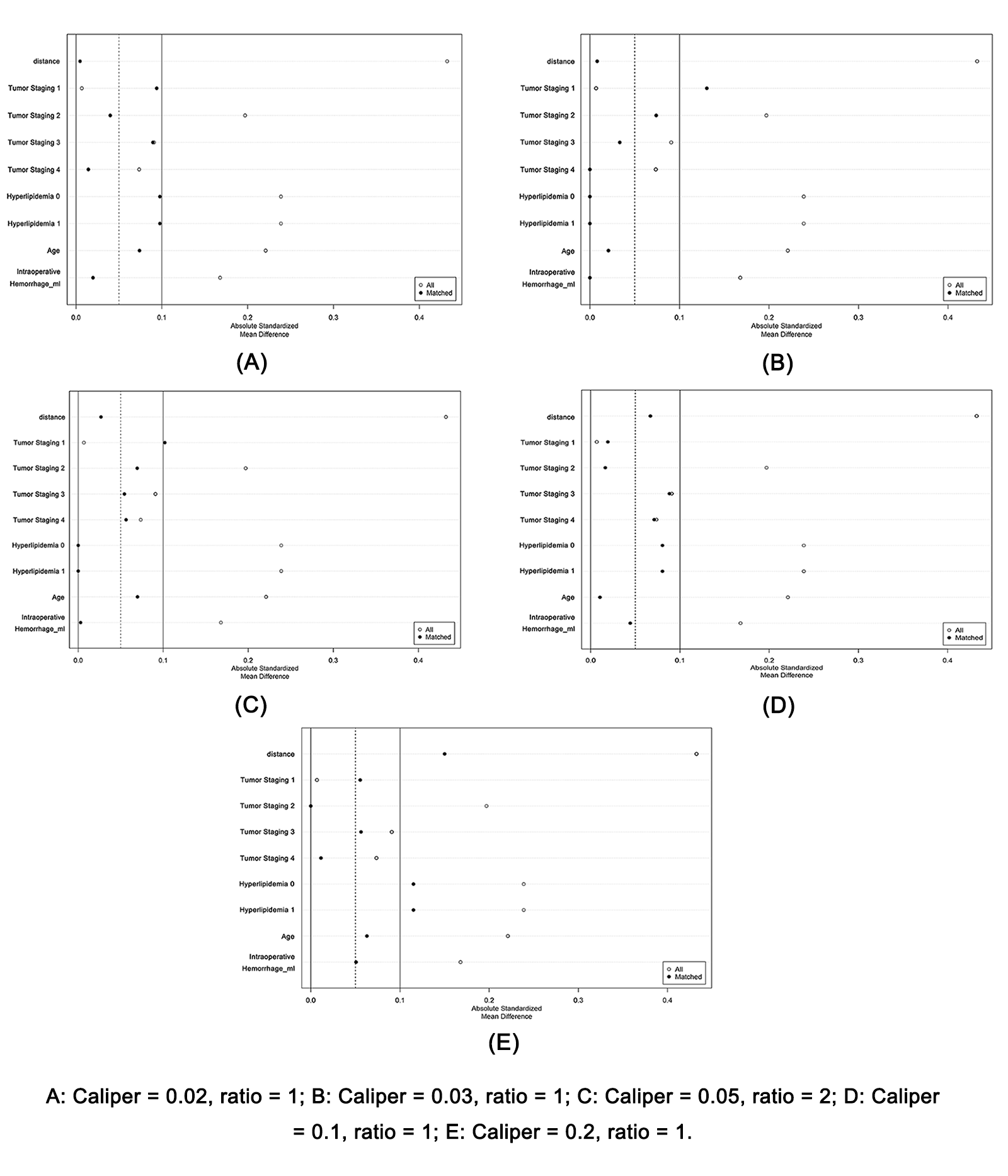

Supplement: S3 Fig — (TIF) [file pone.0303951.s003.tif]

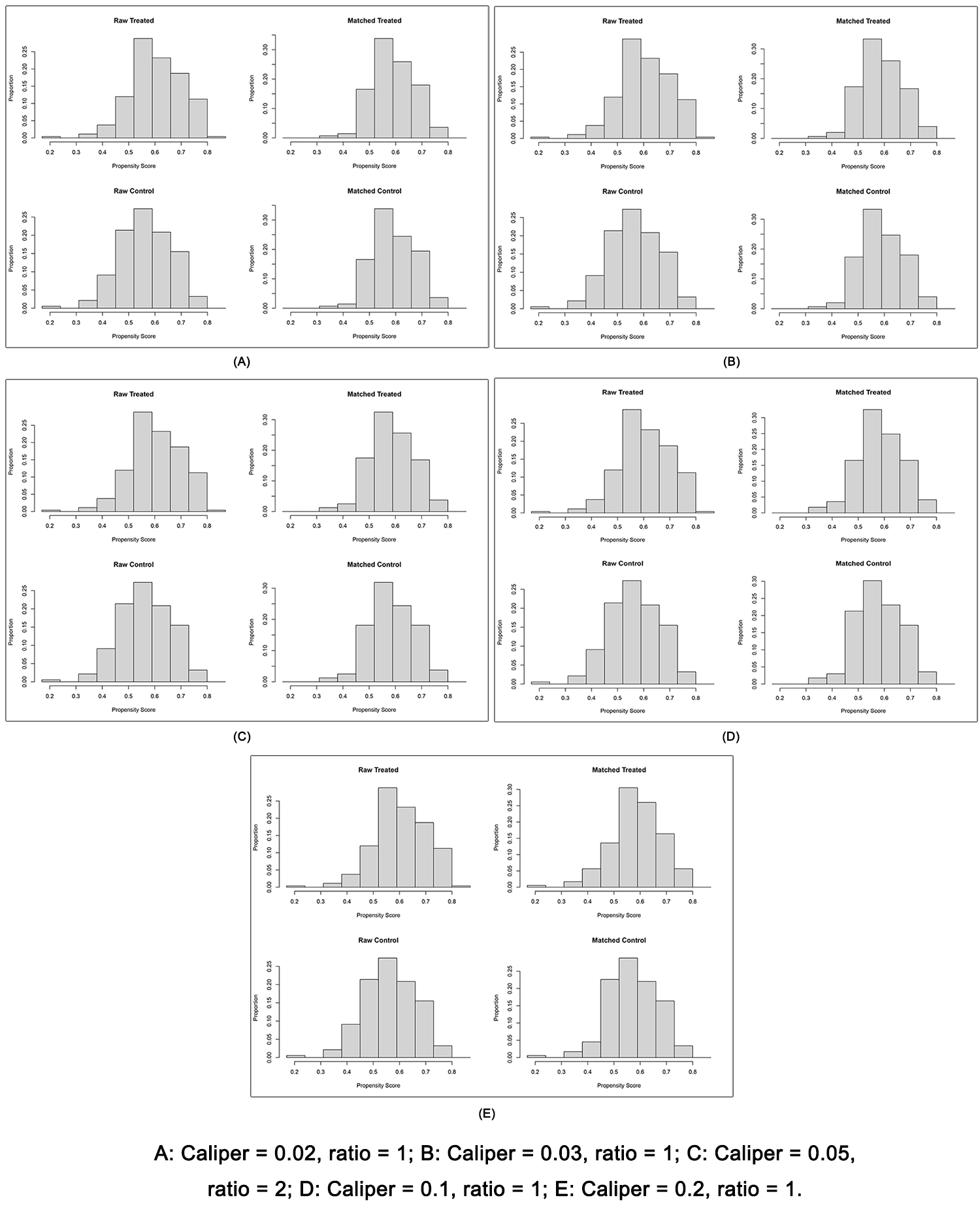

Supplement: S4 Fig — (TIF) [file pone.0303951.s004.tif]
