# Supplementary material for: The effectiveness of Evodia rutaecarpa hot compress on the recovery of gastrointestinal function after laparoscopic surgery for colorectal cancer: A propensity score-matched retrospective cohort study
Source: PLoS One. 2025 Feb 20;20(2):e0303951. doi: 10.1371/journal.pone.0303951 (PMC11841865; doi:10.1371/journal.pone.0303951)
Supplement: S1 Table — (PDF) [file pone.0303951.s005.pdf]

PSM	Variables	95% confidence interval	
		ER group	Non-ER group
Before	Age	(58.12, 60.61)	(60.34, 62.96)
	BMI	(22.48, 23.21)	(22.59, 23.43)
	Hospitalization Expenses_CNY	(74052.73, 76806.53)	(69953.51, 72668.42)
	Hospitalization Days_d,	(16.03, 17.00)	(14.77, 15.79)
	TFFFAS_d	(4.20, 4.69)	(3.82, 4.50)
	TFSFAS_d	(6.68, 7.38)	(6.04, 6.79)
After	Age	(58.17, 61.11)	(58.98,61.71)
	BMI	(22.23, 23.17)	(22.63, 23.53)
	Hospitalization Expenses_CNY	(73106.57, 76606.66)	(68724.58, 71302.86)
	Hospitalization Days_d,	(15.83, 17.09)	(14.47, 15.56)
	TFFFAS_d	(4.01, 4.64)	(3.65, 4.30)
	TFSFAS_d	(6.40, 7.21)	(5.84, 6.57)
