# Supplementary material for: The effectiveness of Evodia rutaecarpa hot compress on the recovery of gastrointestinal function after laparoscopic surgery for colorectal cancer: A propensity score-matched retrospective cohort study
Source: PLoS One. 2025 Feb 20;20(2):e0303951. doi: 10.1371/journal.pone.0303951 (PMC11841865; doi:10.1371/journal.pone.0303951)
Supplement: S2 Table — (PDF) [file pone.0303951.s006.pdf]

Types of postoperative complications	Count
Postoperative nausea and vomiting	9
Postoperative abdominal distension	31
Postoperative occurrence of anastomotic fistula/anastomotic infection	10
Postoperative anastomotic bleeding	4
Postoperative abdominal infection	1
Postoperative intestinal obstruction and paralysis	4

Note: Nine patients simultaneously have two types of postoperative complications.
